# Cytokine Patterns in Maternal Serum From First Trimester to Term and Beyond

**DOI:** 10.3389/fimmu.2021.752660

**Published:** 2021-10-14

**Authors:** Anders Hagen Jarmund, Guro Fanneløb Giskeødegård, Mariell Ryssdal, Bjørg Steinkjer, Live Marie Tobiesen Stokkeland, Torfinn Støve Madssen, Signe Nilssen Stafne, Solhild Stridsklev, Trine Moholdt, Runa Heimstad, Eszter Vanky, Ann-Charlotte Iversen

**Affiliations:** ^1^ Department of Clinical and Molecular Medicine, Norwegian University of Science and Technology (NTNU), Trondheim, Norway; ^2^ Centre of Molecular Inflammation Research (CEMIR), Norwegian University of Science and Technology (NTNU), Trondheim, Norway; ^3^ Department of Public Health and Nursing, Norwegian University of Science and Technology (NTNU), Trondheim, Norway; ^4^ Department of Circulation and Medical Imaging, Norwegian University of Science and Technology (NTNU), Trondheim, Norway; ^5^ Clinical Services, St. Olavs Hospital, Trondheim University Hospital, Trondheim, Norway; ^6^ Department of Obstetrics and Gynecology, St. Olavs Hospital, Trondheim University Hospital, Trondheim, Norway; ^7^ Department of Women’s Health, St. Olavs Hospital, Trondheim University Hospital, Trondheim, Norway

**Keywords:** pregnancy, inflammation, growth factors, colony-stimulating factors, longitudinal cytokine profile, multiplex, reproductive immunology, maternal response

## Abstract

Pregnancy implies delicate immunological balance between two individuals, with constant changes and adaptions in response to maternal capacity and fetal demands. We performed cytokine profiling of 1149 longitudinal serum samples from 707 pregnant women to map immunological changes from first trimester to term and beyond. The serum levels of 22 cytokines and C-reactive protein (CRP) followed diverse but characteristic trajectories throughout pregnancy, consistent with staged immunological adaptions. Eotaxin showed a particularly robust decrease throughout pregnancy. A strong surge in cytokine levels developed when pregnancies progressed beyond term and the increase was amplified as labor approached. Maternal obesity, smoking and pregnancies with large fetuses showed sustained increase in distinct cytokines throughout pregnancy. Multiparous women had increased cytokine levels in the first trimester compared to nulliparous women with higher cytokine levels in the third trimester. Fetal sex affected first trimester cytokine levels with increased levels in pregnancies with a female fetus. These findings unravel important immunological dynamics of pregnancy, demonstrate how both maternal and fetal factors influence maternal systemic cytokines, and serve as a comprehensive reference for cytokine profiles in normal pregnancies.

## 1 Introduction

Pregnancy is a complex and dynamic process during which the immune system must balance tolerance towards the fetus and maternal immunological integrity (1), and serves as a key player for timing and orchestrating normal gestation (2, 3). Studies suggest that the maternal immune system is sensitive to factors such as obesity (4) and fetal sex (5). Aberrant immunological status has been associated with infertility, spontaneous abortions, preterm delivery (6) and a variety of pregnancy-related conditions ranging from perinatal depression (7) to preeclampsia (8). While preterm labor has been studied extensively, less is known about late and post-term pregnancy and labor, even though 4-10% of all pregnancies continue beyond term (9). Late term pregnancy may involve considerable stress for both mother and fetus, but the immunological development has not been characterized. Mapping the staged immunological adaptions throughout pregnancy and the impact of maternal and fetal factors can help mothers and clinicians make informed decisions to reduce the physiological burden of pregnancy and provide the fetus with an optimal outset for life.

It has been postulated that a normal pregnancy comprises three immunological stages that align with the trimesters (1, 10). Implantation and placentation depend on a local inflammatory environment that supports blastocyst adhesion, invasion of the blastocyst and the trophoblasts, and tissue reorganization in the uterine wall. Secondly, a period of fetal growth follows that is thought to be predominantly anti-inflammatory. Finally, labor and parturition involve local and systemic inflammatory mobilization and activation. These immunological processes are tightly regulated by signaling molecules such as cytokines and C-reactive protein (CRP). Cytokines are important for inter-cellular communication at the maternal-fetal interface and are released to maternal blood from the placental surface (11, 12). Combined with cytokines released from blood cells and other tissues, the maternal serum cytokines reflect a concert of immunological processes in the body. The systemic immunological state of an individual can therefore be explored through combined assessment of signaling molecules in peripheral blood (13). However, the coordinated development of serum cytokines throughout normal pregnancies has not been adequately described. Earlier attempts have shown strong impact of pregnancy on serum cytokine levels but resulted in conflicting conclusions, as reviewed by Spence et al. (14). While analysis of few or single cytokines has dominated the literature (14), broad cytokine profiling in pregnancy is feasible (4, 13, 15–21) but these studies have typically employed small study samples or examined only parts of pregnancy.

Maternal serum cytokine profiling can be used to gain detailed information about maternal inflammatory status, fetal stress, and early signs of immunological disturbance (13, 17, 21). To this end, immunological profiling by simultaneous measurement and analysis of multiple cytokines provides higher sensitivity and depicts ongoing inflammatory processes better than single cytokine measurements as cytokines comprise complex functional networks (17, 22–24). We have previously used serum cytokine profiling and multivariate statistics to reveal characteristic changes in cytokine patterns during the first half of pregnancy (13) and demonstrated deviant cytokine patterns before onset of clinical signs in hypertensive pregnancy disorders (21).

In this study we have constructed longitudinal cytokine profiles by analyzing serum from 707 women with normal pregnancies giving birth between week 37^+0^ (37 completed weeks + 0 days) and week 43^+1^. Serum levels of 22 different cytokines and CRP measured by multiplex analysis were used to establish continuous and trimester-specific time trajectories, revealing clear proof of staged immunological adaptions throughout pregnancy. In addition, body mass index (BMI), smoking, parity, fetal sex, and birth weight were shown to be reflected by distinct changes in the maternal serum cytokine profile. This study thus provides a broad and detailed overview of the dynamic maternal immunological status in pregnancy.

## 2 Material and Methods

### 2.1 Study Design

Women with normal singleton pregnancies were selected from five different cohorts (**Supplementary Table 1**). The selection was based on the criteria that they did not have hypertensive or inflammatory conditions or pregnancy complications, such as gestational diabetes or preterm labor, and had provided one or more serum samples during pregnancy (**Supplementary Figure 1**). Pregnancies diagnosed with fetal growth restriction and women with morbid maternal obesity (BMI ≥ 40) or underweight (BMI < 18.5) at inclusion or prior to pregnancy were excluded.

Serum samples were collected between 2002 and 2015 at St. Olavs hospital, Trondheim University Hospital, Trondheim, and between 2003 and 2012 at Haukeland University Hospital, Bergen. Ethical approvals for the specific cohorts cover cytokine profiling and are listed in **Supplementary Table 1**. All participants have given written informed consent.

### 2.2 Serum Cytokine Measurements

Venous blood was drawn from the antecubital vein and transferred to custom serum separator tubes, inverted, kept at room temperature for 30-120 minutes, centrifuged for 10 minutes, and the resulting serum stored at -80°C. The serum samples were analyzed for 27 cytokines (Bio-Plex Pro Human Cytokine 27-plex Assay) in single replicates using Luminex xMAP Technology on a Bio-Plex 200 System (Bio-Rad Laboratories, CA, USA). The cytokines analyzed were eotaxin (eotaxin-1, also known as CCL11), basic fibroblast growth factor (FGF-b, also known as FGF-2), granulocyte (G-) and granulocyte-macrophage (GM-) colony-stimulating factor (GCF), interferon (IFN-) γ, interferon gamma-induced protein (IP-)10 (also known as CXCL10), interleukin (IL-) 1β, IL-1 receptor antagonist (IL-1Ra), IL-2, IL-4, IL-5, IL-6, IL-7, IL-8 (also known as CXCL8), IL-9, IL-10, IL-12p70 (IL-12), IL-13, IL-15, IL-17, monocyte chemotactic protein (MCP-)1 (also known as CCL2), macrophage inflammatory protein (MIP-)1α (also known as CCL3), MIP-1β (also known as CCL4), platelet-derived growth factor (PDGF-BB), RANTES, tumor necrosis factor-α (TNF-α), and vascular endothelial growth factor A (VEGF). The manufacturer’s protocol was followed with the recommended concentration of reagents and serum, but in half the volume (NormalFlow used 1:2.5). This modification has been used in previous studies (13, 21) without reducing assay performance. Serum samples from the included cohorts (except NormalFlow) were distributed across plates using a block design. Each plate contained duplicates of cytokine standards, sample diluent and a pooled control sample made from two women with severe preeclampsia. Serum samples from the NormalFlow cohort was previously analyzed by a similar protocol (13).

High-sensitivity CRP (hsCRP) measurements in serum was obtained using Human CRP Quantikine kit (R&D technologies, MN, USA) in the NormalFlow cohort (13), and a turbidimetric assay and measured at 571 nm by a Roche Modular P analytical system at the Department of Clinical Chemistry at St. Olavs hospital in samples from the Postterm, Preeclampsia (21) and the ETIP studies (25). The TRIP samples were analyzed for hsCRP using turbidimetric assay and measured at 571 nm by a Siemens Advia Chemistry XPT system at the Department of Clinical Chemistry at St. Olavs hospital.

The cytokines were classified into functional groups (13). CRP is an acute-phase protein acting as inflammatory marker and was classified as an inflammatory cytokine for simplicity.

### 2.3 Data Preprocessing

#### 2.3.1 Adjustment of Batch and Plate Effects

In high-throughput technologies such as bead-based immunoassays, results may vary depending on laboratory conditions, lot-specific reagents, and personnel differences. These batch and plate variations must be addressed to avoid effects possibly leading to incorrect conclusions (26). Large-scale cytokine analyses are unfortunately complicated by the lack of standardized methods for batch and plate adjustment (26–28).

The adjustments were first performed for assay *inter*-lot variations and secondly for assay *intra*-lot variations. As advised by Breen et al. (29), the adjustments were performed on fluorescent intensity (FI) values before estimating concentrations from standard curves. Both steps were based on the same expression:


$$\hat{FI_{i}}=\exp\left(\frac{\log(FI_{i})}{\bar{\log{\left(FI\right)}_{R}}}\bar{\log{\left(FI\right)}_{T}}\right)$$

where each term is as follows:

-
$\hat{FI_{i}}$ : Adjusted fluorescence intensity for sample *i*
- *FI_i_*: Unadjusted fluorescence intensity for sample *i*
-
${\bar{\log(FI)}}_{T}$ : the mean of log(*FI_t_*) for samples *t* in a set of reference samples, *T*, representing the target of normalization
-
${\bar{\log(FI)}}_{R}$ : the mean of log(*FI_r_*) for samples *r* in a set of unadjusted reference samples, *R*, that are to be adjusted

The correction was performed for each cytokine separately. The steps are described in detail below together with the step-specific form of the given expression.

##### 2.3.1.1 Correction of Inter-Lot Variations

A reference lot was chosen. From each non-reference lot, eight serum samples were selected before the mean log(*FI*) of these eight samples was calculated. The mean log(*FI*) for lot *j* is denoted ${\bar{\log(FI)}}_{j,0}$ . The eight selected serum samples from each non-reference lot were then included in the reference lot and re-analyzed. The mean log(*FI*) of the eight samples from lot *j* as measured in the reference lot is denoted ${\bar{\log(FI)}}_{j}$ . For each serum sample *i* in the non-reference lot *j*, an adjusted FI value was calculated as


$$\text{adjusted }FI_{i}=\exp\left(\frac{\log(FI_{i})}{{\bar{\log(FI)}}_{j,0}}{\bar{\log(FI)}}_{j}\right)$$

##### 2.3.1.2 Correction of Intra-Lot Variations

Plate-to-plate variations within lots were adjusted for as described by Browne et al. (30). Within each cytokine assay lot, we identified a set of samples, *S*, that was distributed across all plates and that was expected to have equal values on group level. These were samples from a single cohort taken at approximately the same gestational age and randomized across plates. We calculated the mean log(*FI*) for these samples within and across plates and used the difference between within-plate means and across-plate mean to adjust the samples per plate according to the formula


$$\hat{FI_{l}}=\exp\left(\frac{\log(FI_{i})}{{\bar{\log(FI)}}_{j}}\bar{\log(\text{FI})}\right),$$

where *FI_i_* and $\hat{FI_{l}}$ are FI for sample *i* (on plate *j*) before and after correction, respectively, ${\bar{\log(FI)}}_{j}$ the mean log(*FI*) for the selected samples *S* on plate *j*, and $\bar{\log(FI)}$ the mean log(*FI*) for the selected samples *S* across the entire lot.

#### 2.3.2 Estimation of Cytokine Levels

Cytokine concentrations were estimated from FI using cytokine specific calibration curves constructed from standard samples in the reference lot. The nCal package for R (31) was used to calculate concentrations, and limits of detection (LOD) and quantification (LOQ) were estimated from a power model of variance. Non-detectable cytokine values below lower (L-) LOD were imputed using an expectation-maximization algorithm from the zCompositions package in R (32). Cytokine values above upper (U-) LOD were replaced by ULOD. Cytokines with more than 25% above ULOQ or below LLOQ measurements were excluded.

### 2.4 Missing Data and Outlier Detection

Twenty-one serum samples lacked registration of gestational age at sampling. The missing sampling times were substituted by the average gestational age at sampling for similar serum samples in the corresponding cohort. Outliers were identified from visual inspection of the PCA scores by cohort.

### 2.5 Statistical Analysis

Cytokine concentrations were log_e_-transformed unless otherwise specified, to improve the normality of the residuals. Visualizations were made with the ggplot2 package in R (33, 34) version 4.0.2 (35). P values were corrected for multiple testing with the Benjamini-Hochberg procedure (36).

#### 2.5.1 Serum Cytokine Profile From First Trimester to Term

The continuous progression of each cytokine was estimated with generalized additive mixed models (GAMMs) using the gamm4 package for R (37). Generalized additive models are regression models in which coefficients are replaced by smooth functions, in this case penalized cubic regression splines. Untransformed cytokine concentration was modelled with gestational age at sampling and cohort as fixed effects and participant as random effect. To assess the robustness of time trajectories, we repeated the analysis 50 times with stratified random exclusion of 20% of the participants in each run. We then calculated the Spearman’s correlation coefficient between the mean of all runs and each run. Cytokines with an average correlation coefficient below 0.90 were considered unreliable and excluded from the continuous time analysis.

The change in cytokines grouped by trimester was modelled with linear mixed models (LMMs) using the lme4 package for R (38). The serum concentration of each cytokine was log-transformed and modelled with trimester and cohort as fixed effects and participant as random intercept. Statistical significance was calculated with first trimester levels as baseline. Serum samples were classified as first (< week 14^+0^), second (weeks 14^+0^-27^+6^) or third trimester (week 28^+0^-40^+3^), or late term (> week 40^+3^). Correlations between log-transformed cytokine concentrations were calculated separately within each trimester and late term, using Spearman’s correlation coefficient (*ρ*).

#### 2.5.2 Serum Cytokine Profile Beyond Term

In the first late term analysis, the serum cytokine profile in term samples (collected between weeks 37^+0^ and 40^+3^) were compared to late term samples (collected after week 40^+3^). Mann-Whitney’s U test was used to test for significant differences in log concentration. To remove the immediate effect of approaching labor, women with delivery (spontaneous labor, cesarean section, or induction) within four days were excluded and the analysis repeated. In the second late term analysis, late term serum samples were used to build a Fine‐Gray subdistribution hazards model with competing risks (39) to assess overall association with labor. Spontaneous labor was treated as the event of interest. Women randomized to labor induction at week 41^+2^, and not going into spontaneous labor before induction, were treated as censored observations. Labor induction on clinical indication, despite being randomized to monitoring, was treated as a competing event. The cytokine concentrations were log-transformed and normalized to make the hazard ratios comparable. In the third late term analysis, a predictive model was made by orthogonalized partial least-squares discriminant analysis (O-PLS-DA) (40) with spontaneous labor within four days and either spontaneous labor or induction after more than four days as outcomes. The analysis was carried out with autoscaled cytokine levels with the PLS_toolbox 8.9 (Eigenvector Research, WA, USA) in MATLAB 2020a (The MathWorks, Inc., Natick, Massachusetts, United State). Classification error rate, sensitivity and specificity were calculated by building a model from 90% of the participants and predicting the outcome for the remaining participants. Permutation testing (*n* = 1000 permutations) was used to test model quality. For each permutation, the outcomes were shuffled, 90% of the participants were randomly chosen as a training set and used to make an independent model to predict the outcome of the remaining 10% of the participants. The classification error rate was extracted for each permutation and the P value of the model calculated as the proportion of runs with classification error rate lower or equal to the original model.

#### 2.5.3 Modifiers of Maternal Serum Cytokine Profile

A recently developed statistical method, Repeated Measures ASCA+ (RM-ASCA+), was used to assess longitudinal changes and differences between clinical groups (41). RM-ASCA+ is a method for analysis of repeated measures multivariate data, combining traditional univariate statistics of longitudinal data with multivariate dimension reduction techniques. The analysis is performed in two steps: first a separate LMM is constructed for each cytokine, estimating the effect of time and group, and the interaction between time and group for each variable. Second, PCA is performed on the resulting effect matrices yielding component scores and loadings to extract patterns across variables. The time effect and the time-group interaction can be divided into separate effect matrices to separate the variations for visualization (42). Time, group, time-group interaction, and cohort were included as fixed effects and participant as random intercept. Normalization was performed prior to RM-ASCA+ analysis by subtracting the overall mean (across cohorts) by cytokine and dividing by the unweighted mean of the standard deviation per cohort (43). Analyses were performed with the ALASCA package in R (44).

Jackknifing with seven-fold stratified subsets with 100 iterations was used to estimate the robustness of the RM-ASCA+ results. For each iteration, the participants were divided into seven subsets with the relative group sizes preserved in each subset and the analysis performed with one subset excluded. The 2.5^th^ and 97.5^th^ percentiles of results from the jackknifing procedure were plotted as error bars around the estimates based on the full data.

The single serum samples from the late term pregnancies were analyzed separately with linear regression since there was no overlap in sampling time with the other four cohorts. The regressions were performed with log-transformed cytokine concentration normalized by mean centering and division by standard deviation. Maternal age, BMI, and parity were assessed as continuous variables, in contrast to RM-ASCA+ where categorical groups were assessed. For birth weight, z scores were calculated from Norwegian references (45).

## 3 Results

### 3.1 Participants, Serum Samples and Immunoassay Performance

In total 943 pregnant women from five cohorts (**Supplementary Table 1**) were assessed for inclusion to this study (**Supplementary Figure 1**). Of these, 227 women were excluded due to pregnancy complications (*n* = 147), pathological BMI (< 18.5 kg/m^2^, *n* = 6; or ≥ 40 kg/m^2^, *n* = 8) or missing serum samples (*n* = 66). Hypertension was the most common reason for exclusion (*n* = 115 women). Nine additional women were excluded as outliers by visual inspection of principle component analysis (PCA) scores (**Supplementary Figure 2**), leaving a final set of 707 women (**Supplementary Figure 1**). Maternal pre-pregnancy or first trimester BMI was distributed among the women as 64% normal weight (18.5-24.9 kg/m^2^), 26% overweight (25.0-29.9 kg/m^2^) and 10% obesity (≥ 30.0 kg/m^2^) (46). Of the included women, 10% were registered as smokers at inclusion, 46% were nulliparous, and 42% were carrying a female fetus. At birth, 12% of the neonates were below the 25^th^ percentile for expected birth weight, 60% were between the 25^th^ and 75^th^ percentile, and 28% were above the 75^th^ percentile. The pregnant women were divided into two study groups reflecting the gestational age at serum sampling: a term study group (serum sampled at or before gestational week 40^+3^, *n* = 313) and a late term study group (serum sampled after gestational week 40^+3^, *n* = 396) (**Table 1**). In total, 1149 serum samples were included. The 753 serum samples in the term study group were distributed throughout gestation with 192 (25%) collected in first trimester, 367 (49%) in second trimester, 194 (26%) in third trimester; 84% of the women participated with more than one serum sample. All women in the late term study group had only a single serum sample included.

**Table 1:** Clinical characteristics of the two study groups (*n* = 707).

	Term^*^	Late term
*n*	311	396
Maternal age (years)	29.6 (4.4)	30.3 (4.7)^3^
Body mass index, (kg/m^2^)^**^	25.1 (4.1)^30^	24.4 (3.5)
Gestational age at delivery (days)	280.2 (8.8)^14^	291.7 (2.6)
Birth weight (grams)	3572 (438)^22^	4015 (428)
Fetal sex, female, *n* (%)	146 (50)^18^	146 (37)
Smoking (yes), *n* (%)	17 (6)^40^	48 (12)
Nulliparous, *n* (%)	154 (50)^4^	167 (42)

^*^The term study group was composed of women from four cohorts (**Supplementary Table 1**). ^**^BMI was measured at inclusion in three of the cohorts comprising the term study group. Pre-pregnancy BMI was used for the fourth cohort comprising the term study group and for the late term study group. Data are listed as mean (standard deviation)^m^ or number n (%)^m^, where m is the number of missing data points. The term study group included women with serum sampled at or before week 40^+3^ of gestation and the late term study group included women with serum sampled after week 40^+3^ of gestation.

The cytokines IFN-γ, RANTES, VEGF and IL-10 had high proportions of non-detectable values (either below LLOQ or above ULOQ in > 25% of the samples) in the cytokine multiplex assay on maternal serum and were excluded (**Supplementary Table 2**). IL-5 was excluded due to high proportion of missing values in one cohort. The remaining 22 cytokines were within the limits of quantification in at least 75% of the serum samples.

### 3.2 Serum Cytokine Profile From First Trimester to Term

**Figure 1:**
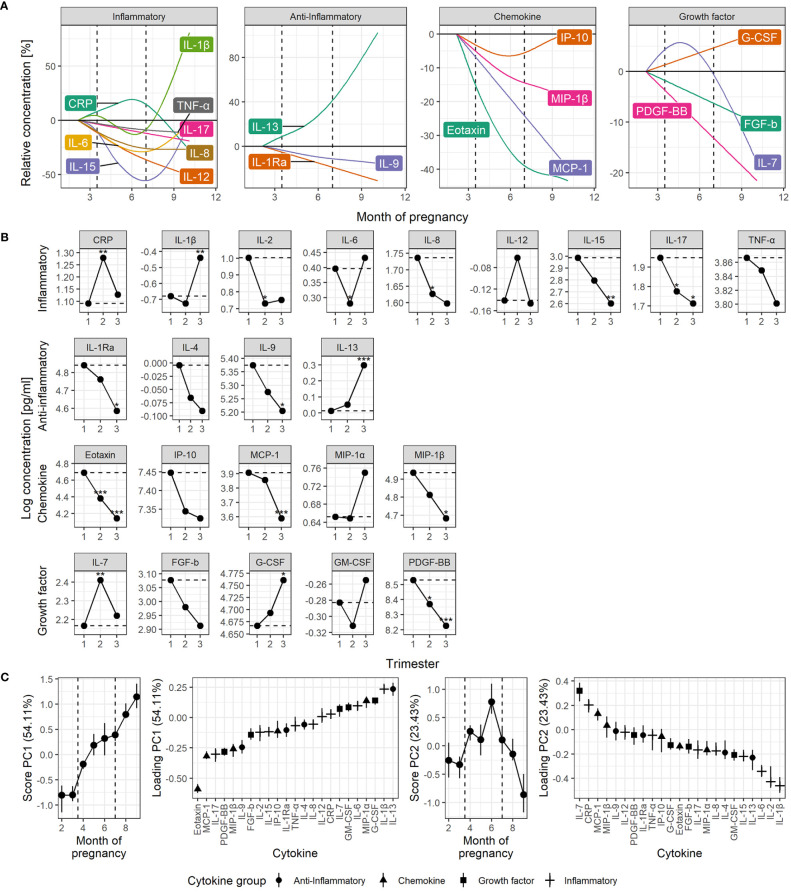
Time course of serum cytokine levels throughout normal pregnancy. **(A)** Cytokine levels by functional group, shown relative to early pregnancy. Trajectories are estimated by generalized additive linear mixed models. The dashed lines indicate the trimesters. **(B)** Cytokine levels shown by trimester estimated by univariate linear mixed models. Asterisks indicate significant change from the first trimester (dashed line). **(C)** RM-ASCA+ analysis of the longitudinal serum cytokine profile. Loadings (right panels) describe the contribution of each cytokine to the corresponding scores, representing the overall cytokine changes throughout pregnancy (left panels). Higher score translates to higher levels of cytokines with positive loading and lower levels of cytokines with negative loading, and vice versa. Error bars from jackknife validation. The first principal component (PC1) summarizes the most common trends and explains 54% of the cytokine variation over time. Some cytokines have a development not well described by PC1, requiring a separate trajectory (PC2). CRP is measured in µg/mL, cytokines in pg/mL. *P < 0.05, **P < 0.01, ***P < 0.001.

Quantification of individual serum cytokine development from first trimester to term revealed diverging trajectories within all four groups of cytokines (**Figure 1**). The construction of robust, continuous trajectories was achievable for 19 of the 22 cytokines, and CRP (**Figure 1A** and **Supplementary Figure 3**). Four common patterns of cytokine development emerged: (1) continuous increase, (2) continuous decrease, (3) non-linear progression with maximal peak in the second trimester, and (4) non-linear progression with dip in the second trimester (**Figure 1A**). For most cytokines, the highest serum level occurred in the first trimester and 11 cytokines decreased significantly from the first to the second and/or third trimester (**Figure 1B**). Seven cytokines showed no significant change from first to subsequent trimesters (**Figure 1B**).

Assessment of the cytokine levels continuously over time revealed that most inflammatory cytokines peaked in the first trimester, decreased in the second trimester, and subsequently increased again towards the end of the third trimester, and most potently for IL-1β (**Figure 1A**). A significant decrease in inflammatory cytokine levels from the first to the second trimester was evident for IL-2, IL-6, IL-8, and IL-17 (**Figure 1B**). CRP clearly deviated from the inflammatory cytokine pattern by demonstrating a peak in the second trimester (**Figure 1B**) and was only weakly correlated to cytokine levels in general (**Supplementary Figure 4**). IL-6, a well-known inducer of CRP, showed moderate correlation with CRP in the second (*ρ*=0.33, *P *< 0.001) and third trimester (*ρ*=0.33, *P* < 0.001), and in late term (*ρ*=0.23, *P *< 0.001), but not in the first trimester (*ρ*=0.18, *P* = 0.085).

The anti-inflammatory cytokines showed diverse development (**Figure 1A**). IL-1Ra and IL-9 decreased slowly but steadily throughout pregnancy while IL-13 surged towards term (**Figure 1B**). The growth factors also showed diverse trajectories. IL-7 peaked in the second trimester and G-CSF increased steadily throughout pregnancy in contrast to PDGF-BB that decreased (**Figure 1B**). The chemokines eotaxin, MCP-1 and MIP-1β decreased from first trimester to term (**Figure 1B**), and clearly most pronounced for eotaxin (**Figures 1A, B**).

The contribution of each cytokine to the gestational changes were assessed by multivariate RM-ASCA+ analysis. This overall approach visualized the four common trajectories of serum cytokine development (**Figure 1C**) and clearly supported the longitudinal development of separate cytokines (**Figure 1B**). Steady increase or decrease, represented by the first principal component (PC1), explained more than half the variation in cytokine levels throughout pregnancy and was especially prominent for eotaxin (**Figure 1C**). Thirteen of the cytokines decreased throughout pregnancy, as shown by their robust, negative loading on PC1, whereas seven showed an overall increase. Additional variation in cytokine levels over time identified a particularly strong contribution from the inflammatory cytokines IL-1β, IL-2 and IL-6, with the lowest levels during the second trimester followed by an increase towards term, as explained by a non-linear component (PC2, **Figure 1C**). IL-7 and CRP showed the opposite trend and peaked in the second trimester before decreasing towards term. IL-1β and IL-13 showed a strong surge in the third trimester, by their positive loadings on both PC1 and PC2 (**Figure 1C**). Cytokine levels were mainly positively correlated within all three trimesters and the highest number of positive correlations was observed in late term. In contrast, the highest number of negative correlations were clearly apparent in the second trimester (**Supplementary Figure 4**).

### 3.3 Serum Cytokine Profile Beyond Term

**Figure 2:**
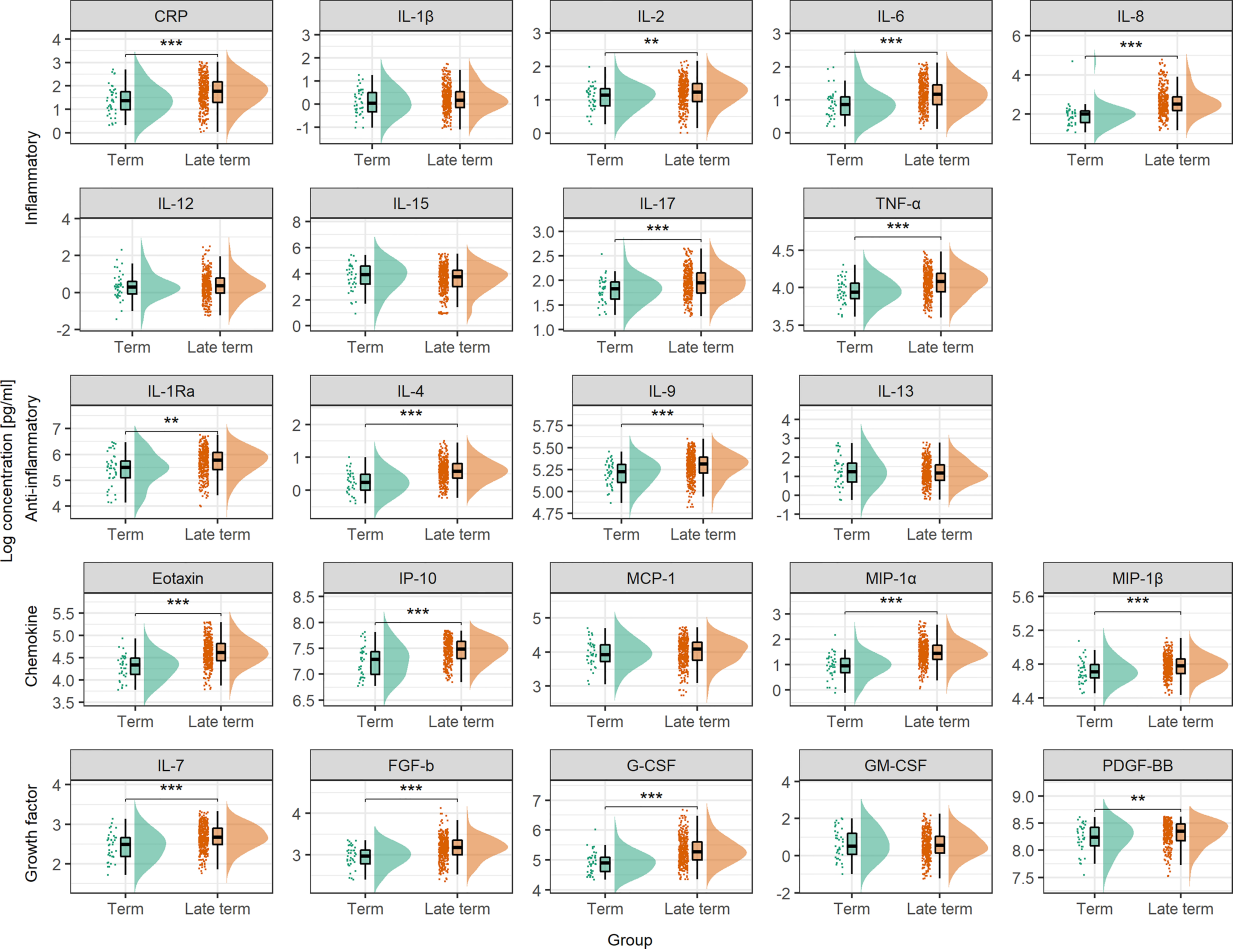
Serum cytokine concentration at term and in late term. Serum cytokine concentrations are compared between term samples taken between week 37^+0^ and 40^+3^ of pregnancy (*n* = 44) and late term samples taken at approximately week 41^+2^ (*n* = 396). Concentrations are shown as individual measurements, median and quantiles as box plot, and as distribution. To enhance readability, percentiles below 2.5 and above 97.5 are not shown. Statistical significance from Mann-Whitney’s U test. CRP is measured in µg/mL, cytokines in pg/mL. **P < 0.01, ***P < 0.001.

Progressing beyond term (40^+3^ weeks) had potent effect on the maternal serum cytokine profile with significantly higher serum levels of as many as 16 of the 22 multifunctional cytokines, and CRP, in late term compared to term pregnancies (**Figure 2**). The coordination of this cytokine boost was apparent by the higher number of positively correlated cytokines in late term serum compared to term (**Supplementary Figure 4**). To separate the effect of progressing pregnancy from approaching labor, only term and late term pregnancies with more than four days left until normal delivery were compared, and cesarean sections (n = 22 term) and induced deliveries (n = 1 term and n = 200 late term) within four days were excluded. The effect of progressing pregnancy beyond term was even stronger in absence of labor, with significantly higher serum levels of all cytokines except FGF-b (**Supplementary Figure 5**).

The late term study group provided the opportunity to elaborate how serum cytokines were affected by approaching labor. In this group, 169 women went into spontaneous labor and 227 had induced labor due to randomization or clinical indication (47). The women with spontaneous labor had an average delay of 3.4 (SD = 2.5) days from serum sampling to delivery.

**Figure 3:**
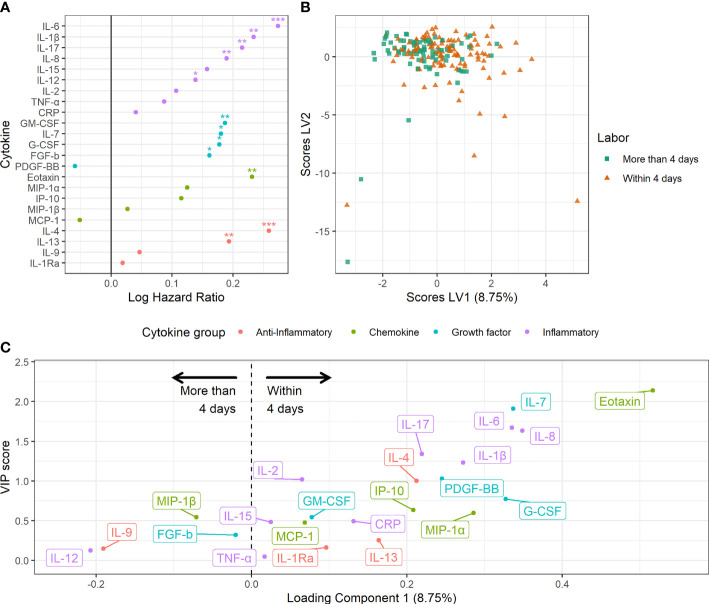
Maternal serum cytokine profiles in late term pregnancies approaching labor. **(A)** Association between serum cytokine levels and risk hazard ratio for spontaneous labor. Estimation from Fine and Gray’s proportional subdistribution hazards regression model in late term women. *P < 0.05, **P < 0.01, ***P < 0.001. **(B)** Orthogonalized PLS-DA scores for women with spontaneous labor within four days and women with spontaneous labor or induction after more than four days. **(C)** Variable loadings for the PLS-DA model. Cytokines with positive loadings were increased in pregnancies with spontaneous labor within four days. Higher variable of importance for projection (VIP) scores correspond to higher importance for separation.

Short time to spontaneous labor was reflected by marked and distinct changes in the maternal serum cytokine pattern (**Figure 3**). Overall, shorter time to spontaneous labor was associated with higher serum levels of the inflammatory cytokines IL-1β, IL-6, IL-8, IL-12 and IL-17, the anti-inflammatory cytokines IL-4 and IL-13, the chemokine eotaxin and the growth factors FGF-b, IL-7, G-CSF and GM-CSF (**Figure 3A**). A multivariate model built to predict spontaneous labor within four days showed low but significant discrimination between the groups (accuracy 57%, sensitivity 60% and specificity 43%, *P* < 0.001) (**Figures 3B, C**). The cytokines with variable of importance for projection (VIP) scores above one were considered most important for predicting spontaneous labor within four days (**Figure 3C**). This revealed that higher serum levels of eotaxin, IL-7, IL-6, IL-8, IL-17, IL-1β, IL-2, PDGF-BB, and IL-4 were associated with spontaneous labor within four days.

### 3.4 Modifiers of Maternal Serum Cytokine Profile

**Figure 4:**
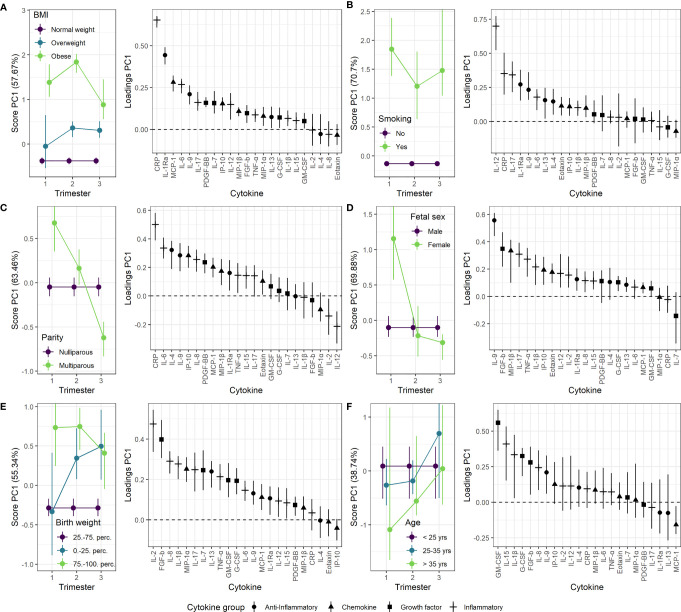
Impact of clinical parameters on the maternal serum cytokine profile from first trimester to term. Serum cytokine profiles throughout pregnancy for **(A)** overweight and obese women relative to normal weight women, **(B)** smoking compared to non-smoking women, **(C)** multiparous compared to nulliparous women, **(D)** pregnancies with a female fetus compared to male fetus, **(E)** pregnancies with birth weight above the 75^th^ percentile or below the 25^th^ percentile of expected sex-specific birth weight compared to those within 25^th^-75^th^. percentile, and **(F)** mothers between 25-35 years and above 35 years of age compared to mothers below 25 years of age. Loadings (right panels) describe the contribution of each cytokine to the corresponding scores (left panels). Higher score translates to higher levels of cytokines with positive loading and lower levels of cytokines with negative loading, and vice versa. The scores describe relative differences between groups and must be interpreted in relation to corresponding time trajectories (**Figure 3**).

The maternal serum cytokine development throughout pregnancy may be influenced by genetics, lifestyle, and other factors. RM-ASCA+ allows for overall separation of time effects from group effects. **Figure 4** shows how clinically important subgroups *differ* from a relevant reference group in longitudinal serum cytokine development.

**Figure 5:**
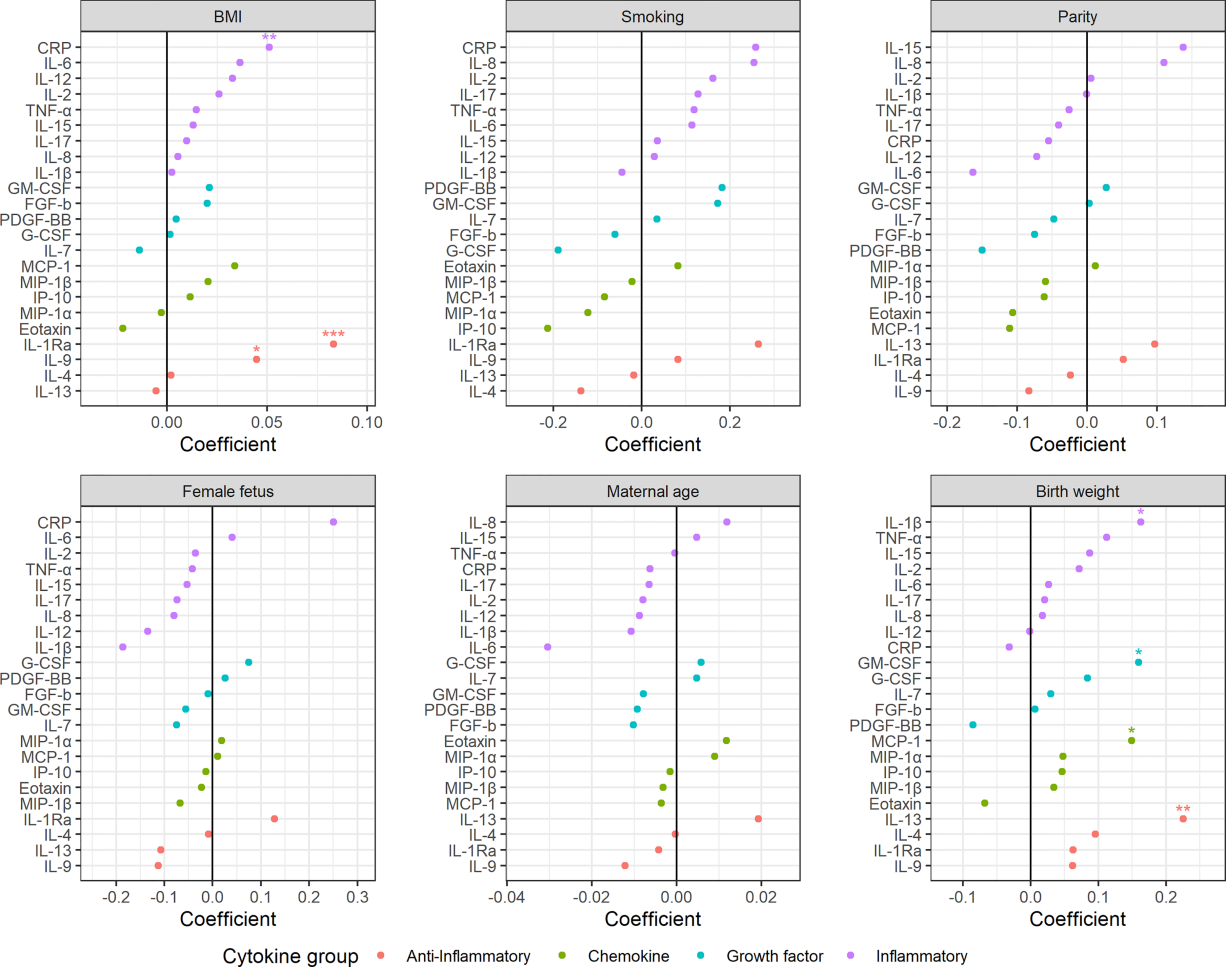
Impact of clinical parameters on the maternal serum cytokine profile beyond term. BMI, parity, maternal age, and birth weight (z-scores) are continuous variables, whereas smoking and fetal sex are categorical variables with non-smoking and female fetuses as reference, respectively. Regression coefficients from linear models. *P <0 .05, **P < 0.01, ***P < 0.001.

Maternal obesity was characterized by increased levels of most cytokines in all three trimesters (**Figure 4A**). The most pronounced increase was found for CRP and IL-1Ra with the highest effect in the second trimester. This relation was further confirmed in late term pregnancies, with significantly higher levels of CRP, IL-1Ra and IL-9 associated with higher pre-pregnancy BMI (**Figure 5**). Overweight pregnant women showed a similar but less pronounced increase of the same cytokines as the obese pregnant women (**Figure 4A**).

Mothers reporting smoking at inclusion showed increased serum levels of both inflammatory and anti-inflammatory cytokines throughout pregnancy compared to non-smokers (**Figure 4B**). The inflammatory cytokines IL-12, IL-17, and CRP showed the strongest contribution to the smoking specific cytokine pattern (**Figure 4B**). No significant effect of smoking was apparent in late term pregnancies (**Figure 5**).

Multiparous women presented with stronger immunological activity in the first trimester; for nulliparous women this effect was completely reversed to stronger immunological activity in the third trimester (**Figure 4C**). CRP and IL-6 contributed the most to this trimester-specific difference depending on parity. No significant effect of parity was observed in late term pregnancies (**Figure 5**).

Women carrying a female fetus showed higher serum cytokine levels in the first trimester compared to pregnancies with a male fetus (**Figure 4D**), and with the strongest contribution to this difference from the anti-inflammatory cytokine IL-9. No such effect of fetal sex was found in late term (**Figure 5**).

Pregnancies with a fetus above the 75^th^ percentile of expected sex-specific birth weight (48) exhibited increased cytokine levels throughout pregnancy, with the strongest contribution from IL-2 and FGF-b (**Figure 4E**). Pregnancies with a fetus below the 25^th^ percentile of expected sex-specific birth weight showed a raise in cytokine levels, especially IL-2 and FGF-b, in the second and third trimester (**Figure 4E**). In late term pregnancy, higher birth weight was associated with higher levels of IL-13, IL-1β, MCP-1 and GM-CSF (**Figure 5**).

Maternal age was not associated with robust changes in cytokine profile, either in term or in late term pregnancies (**Figures 4F** and **5**).

## 4 Discussion

This study tracked the serum levels of 22 cytokines and CRP throughout normal pregnancy and revealed the impact of gestation and clinical variables on the serum cytokine profile. Inflammatory cytokine levels were highest in the beginning and end of pregnancy, except for CRP that showed an opposite pattern. Anti-inflammatory cytokines, chemokines and growth factors followed diverse trajectories and mostly with highest serum levels in the first trimester. A robust decrease of eotaxin throughout pregnancy was identified. Pregnancies continuing beyond term showed a broad serum cytokine boost and specific cytokines increased even more when approaching labor. Interestingly, maternal obesity, smoking, parity, fetal growth and sex induced unique cytokine signatures. In contrast to the sustained activation from obesity and smoking, parity and fetal sex had trimester-specific effects on the serum cytokine profile. Contrary to serum cytokines, CRP was regulated in a less specific manner by most parameters.

Our findings demonstrate that the “immune clock of pregnancy” (2, 3, 49) is accurately reflected in the maternal serum cytokine profile and consistent with the proposed three-staged model of pregnancy with trimester-specific immunological profiles (1, 10). The first trimester presented high levels of most cytokines, including promoters of angiogenesis such as IL-2, IL-8, IL-17, MCP-1 and eotaxin (50–53). The second trimester was characterized by reduced levels of inflammatory cytokines, consistent with an anti-inflammatory period focused on fetal growth (1, 10). CRP posed an important exception with peak levels in the second trimester and this has been corroborated by others (54–56) while contrasted by smaller studies reporting no consistent change (57, 58), or increasing levels throughout pregnancy (59). The second trimester also featured the lowest number of correlating cytokine levels, possibly as result of a conflict between regulation and stress. Finally, the third trimester introduced an immunological mobilization, probably as prelude for the inflammatory processes involved in labor but also as sign of increased burden and stress (1, 10). Late term pregnancies showed a strong increase in most cytokine levels, suggesting added stress when pregnancy progresses beyond term and warranting caution for potential impact of such immune activation. The profound shifts in cytokine profile at different stages of pregnancy indicate that the systemic immune status mirrors gestational processes.

Cytokines are multifunctional and may act in different ways depending on context. High CRP and low inflammatory cytokine levels were characteristic for the cytokine profile in the second trimester. CRP is best known as an inflammatory marker, but increased CRP levels in the absence of other inflammatory markers (IL-1β, TNF-α) is suggested to contribute to wound healing and tissue repair and having anti-inflammatory effects by inducing M2 phenotype in macrophages (60). A role for CRP in this context supports the anti-inflammatory state of second trimester (1, 10) and warrants caution for using CRP alone to monitor inflammation in pregnancy. IL-7 also peaked in the second trimester and has been suggested important for maintaining fetal tolerance through the induction of δγ T cells and uterine NK cells (61). Previously, increasing (4) or stable (15, 62) levels of IL-7 have been reported, but then measured at different time points in pregnancy. Special attention must be drawn to the strong expression of the chemokine eotaxin in early pregnancy and the marked decrease towards term, as supported by others (4, 15). The role of eotaxin in pregnancy is largely unknown, but *in vitro* experiments support importance of eotaxin in first trimester by its involvement in extravillous trophoblast invasion and migration (63). Importantly, the lack of impact from clinical parameters on the eotaxin development points to decreasing eotaxin as a robust and reliable hallmark of normal pregnancy. The continuous time analysis captured within-trimester variations that may be missed when samples are pooled in trimesters; these continuous trajectories revealed a u-shaped development for several cytokines, including IL-6. The literature is conflicting and reports both decreasing, increasing and stable IL-6 concentrations throughout pregnancy (14), probably due to methodic choices that did not capture the diverse non-linear regulation of IL-6 shown here. Future studies should take the rapid dynamics of pregnancy into account, especially towards the end of gestation.

Both maternal and fetal factors had unique impact on the serum cytokine profiles. To our knowledge, this is the first study describing the impact of such factors on a broad panel of serum cytokines in a large study group. Higher maternal BMI was associated with higher immune activation throughout pregnancy as reflected by increased cytokine levels in general. The strongest increase, represented by CRP, IL-1Ra, IL-6 and MCP-1, is corroborated by others (20, 64–66). In contrast to the effect of BMI, maternal smoking had less effect on growth factors and chemokines. Smoking reduces the risk of gestational hypertension and mild preeclampsia (67, 68), but increases the risk of fetal growth restriction and preterm delivery (69). The smoking-associated increase in serum IL-17 shown here may relate to the potential role for IL-17 in pregnancies complicated by placental insufficiency (70) or preterm labor (71), and increased IL-17 has been shown in non-pregnant smokers (72). Smoking status was recorded only at inclusion in this study, thus the effect of continued smoking on the maternal immune status in pregnancy should be investigated further. Obesity and smoking impact serum cytokines outside pregnancy (73, 74), and the sustained increase in cytokine levels throughout pregnancy may in part reflect differences in pre-pregnancy immune status. The trimester-specific effects of parity point to stronger immune activation for multiparous women in the first trimester and for nulliparous women in the third trimester. The increased CRP in multiparous women in early pregnancy has been shown previously (75). Our findings substantiate the notion that distinct first trimester cytokine profiles of inflammatory character reflect immunological milieus beneficial for placentation and fetal growth (19). The fact that use of anti-inflammatory drugs in early pregnancy increases the risk of spontaneous abortion (76) supports this assumption. In contrast, the immune mobilization in nulliparous women in the third trimester may relate to the increased levels of IL-4 (14), IP-10 (77), IL-6 and CRP (78) in preeclampsia, since primiparous women have more than doubled risk of developing preeclampsia (79). The lower inflammatory activation in multiparous women in the third trimester, on the other hand, corresponds to their decreased risk for preterm labor (80). We found that carrying a female fetus was associated with increased cytokine levels in the first trimester, supporting the notion of sexual dimorphism in the immunological adaptions of pregnancy (81). Male fetuses seem to elicit a maternal cytokine profile with a stronger inflammatory contribution than pregnancies with female fetuses that are characterized by increased levels of regulatory cytokines (5, 82). The importance of maternal serum IL-9 in this fetal sex dependent difference has been reported (82), while others have not found influence of fetal sex on maternal serum cytokine levels (83). IL-9 can induce immunological tolerance and contribute to the resolution of inflammation, but its role in pregnancy is not established and warrants further investigation (84). Pregnancies with the largest fetuses showed increased cytokine levels starting as early as in the first trimester, while the smallest fetuses showed a similar cytokine pattern gradually emerging in the second trimester. FGF-b in maternal serum has been associated with birth weight in diabetic mothers (85), and suggested to play a role in maternal glycemic control (86). In the placenta, FGF-b may contribute to trophoblast proliferation and vessel formation (87). Thus, high levels of FGF-b as early as in first trimester may contribute to prosperous placentation and reflect a beneficial environment for fetal growth. Low cytokine levels in early first trimester have previously been associated with small-for-gestational-age neonatal outcome (19). The subsequent rise in cytokine levels observed in the current study in pregnancies resulting in low-weight neonates may thus be a response to stress or mild placental insufficiency.

Our exploration of the marked and broad cytokine mobilization in late term pregnancy demonstrates that both the strain of advancing pregnancy and the physiological preparation for labor must be considered as sources of immunological stress. The sensitivity of cytokine profiling thus allows for identification of such stress within the normal range and in absence of other clinical signs. The strong cytokine mobilization of late term pregnancy seemed to surpass and overshadow the influence of clinical parameters such as BMI, but the patterns were still consistent with the observations in term pregnancy. Although late term pregnancies presented a significant maternal serum cytokine boost, short time to labor elevated this response even further, most probably reflecting the late-stage immunological adaptions to the inflammatory scenario of labor and delivery. These findings presented the novel possibility of predicting spontaneous labor by monitoring serum cytokines, but despite the magnitude of the cytokine response close to labor, the prediction model showed low classification rate. One reason may be the seemingly chaotic cytokine expression and immunological regulation in late term pregnancies. In contrast to our results of cytokine upregulation in late term pregnancies, decreased or unchanged levels of IL-6, IL-8 and TNF-α has been associated with prolonged pregnancy (88), including all pregnancies beyond 40^+0^ weeks. However, IL-8 was related to the induction of labor, consistent with our findings (88). Most of the cytokines of high importance to the prediction model have been related to preterm labor in earlier studies (89), indicating that this cytokine pattern is associated with labor regardless of when in pregnancy the labor takes place.

This comprehensive study of maternal serum cytokine profiles establishes an important reference for future research on normal and pathological cytokine patterns in pregnancy due to the large number of participants. The combination of several cohorts with distinct characteristics strongly increased the generalizability of our findings and the potential variations were adjusted for in the sensitive multivariate statistical models. The two study groups (term and late term) were analyzed separately but yielded similar patterns, suggesting valid and reliable biological effects. Although our study was not originally designed to test the predictive power of serum cytokines for time to labor, we found strong indications of such connection with possible clinical utility. We are unable to distinguish the relative contribution from pre-pregnancy (73, 74) and pregnancy specific cytokine levels (4) in the first trimester. We therefore encourage future studies to extend the presented time development to pre-pregnancy, and also to include parameters related to pregnancy complications.

In summary, we have characterized trimester-specific serum cytokine profiles as a sensitive measure of maternal immune status and showed how maternal BMI, smoking, parity, and fetal sex and birth weight affected the immunological development during normal pregnancy. Maternal obesity and smoking were associated with sustained increase in cytokine levels throughout pregnancy, possibly representing increased immunological stress. A steady decrease in eotaxin levels throughout gestation was identified as a reliable hallmark of normal pregnancy. These findings verify the immunological delicacy and fluctuations of normal pregnancy and establish a comprehensive reference for longitudinal cytokine development in pregnancy. Importantly, we identify a probable strain of advancing pregnancy and combined with the physiological preparation for labor, these must be considered as sources of immunological stress in late pregnancy. Serum cytokine profiling provides an accessible and sensitive way of exploring the immunophysiology of pregnancy and may help decode some of the remaining mysteries of human becoming.

## Data Availability Statement

The raw data supporting the conclusions of this article will be made available by the authors, without undue reservation.

## Ethics Statement

The studies involving human participants were reviewed and approved by REK Midt, Trondheim, Norway. The participants provided their written informed consent to participate in this study.

## Author Contributions

EV and A-CI supervised the study and GG provided statistical supervision. Serum samples were collected and provided by BS, SSta, SStr, TMo, RH, and A-CI. AJ, GG, MR, BS, LS, and TMa developed methodology and AJ conducted the formal analyses. AJ and A-CI wrote the manuscript. All named authors contributed significantly to the submitted work, and reviewed and approved the manuscript.

## Funding

This study was supported by funding from the Joint Research Committee (FFU) between St. Olavs Hospital HF and the Faculty of Medicine and Health Sciences, NTNU; the Liaison Committee between NTNU and the Central Norway Regional Health Authority; and the Research Council of Norway through its Centres of Excellence funding scheme, project number 223255.

## Conflict of Interest

The authors declare that the research was conducted in the absence of any commercial or financial relationships that could be construed as a potential conflict of interest.

## Publisher’s Note

All claims expressed in this article are solely those of the authors and do not necessarily represent those of their affiliated organizations, or those of the publisher, the editors and the reviewers. Any product that may be evaluated in this article, or claim that may be made by its manufacturer, is not guaranteed or endorsed by the publisher.

## Acknowledgments

We thank Kirsti Krohn Garnæs and the Clinical Research Facility at St. Olavs Hospital for contributing to serum sampling.
