# Supplementary material for: Cytokine Patterns in Maternal Serum From First Trimester to Term and Beyond
Source: Front Immunol. 2021 Oct 14;12:752660. doi: 10.3389/fimmu.2021.752660 (PMC8552528; doi:10.3389/fimmu.2021.752660)
Supplement: Supplementary file 1 [file Image_1.pdf]

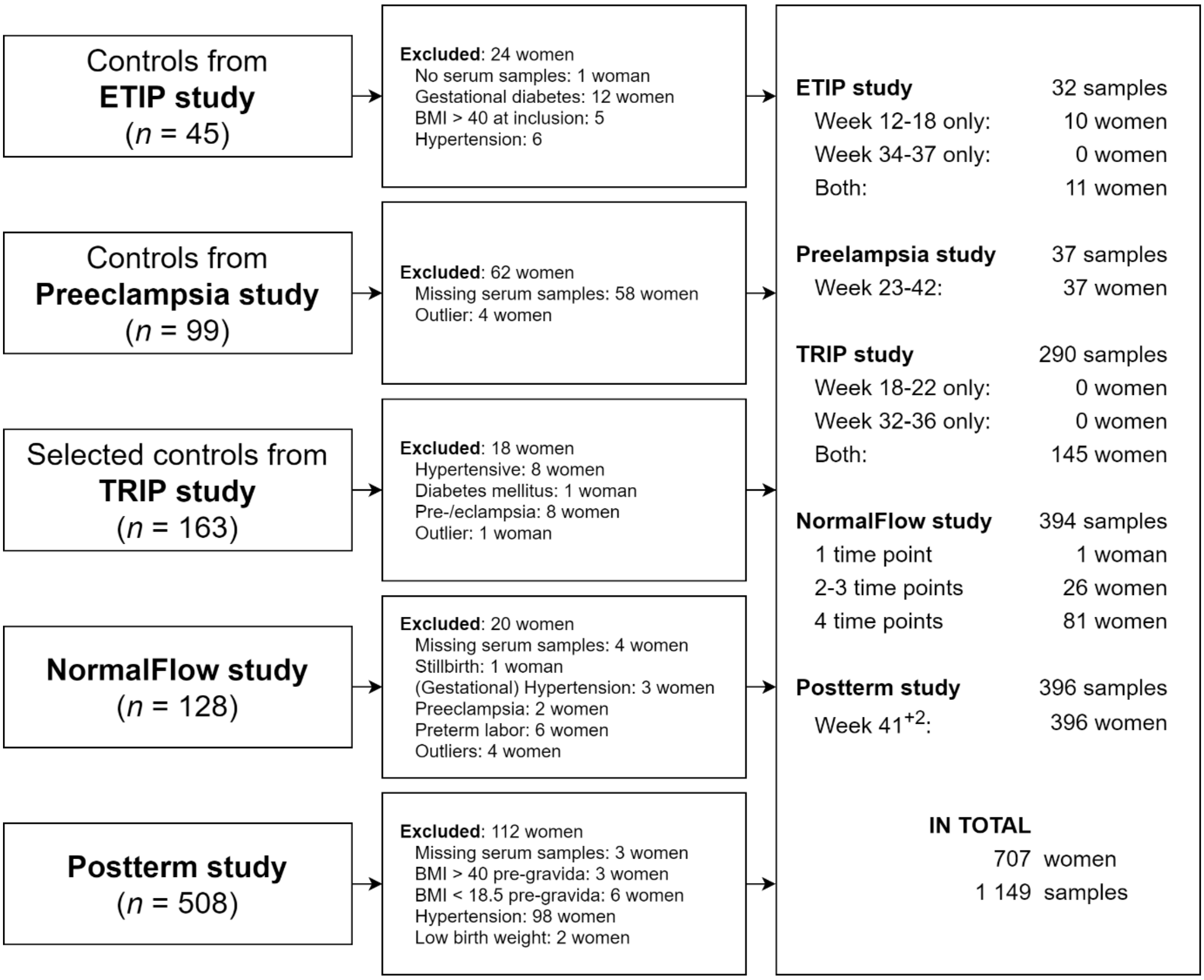

Supplementary Figure 1. **Flow chart of participant selection from five original cohorts.** In total, 943 women were assessed for inclusion as indicated, resulting in 707 unique women and 1149 serum samples included for analysis. Controls from the TRIP study were randomly selected from a larger pool of healthy control participants. ETIP, Exercise Training in Pregnancy for obese women. TRIP, Training in pregnancy.
