# Supplementary material for: Cytokine Patterns in Maternal Serum From First Trimester to Term and Beyond
Source: Front Immunol. 2021 Oct 14;12:752660. doi: 10.3389/fimmu.2021.752660 (PMC8552528; doi:10.3389/fimmu.2021.752660)
Supplement: Supplementary file 2 [file Image_2.pdf]

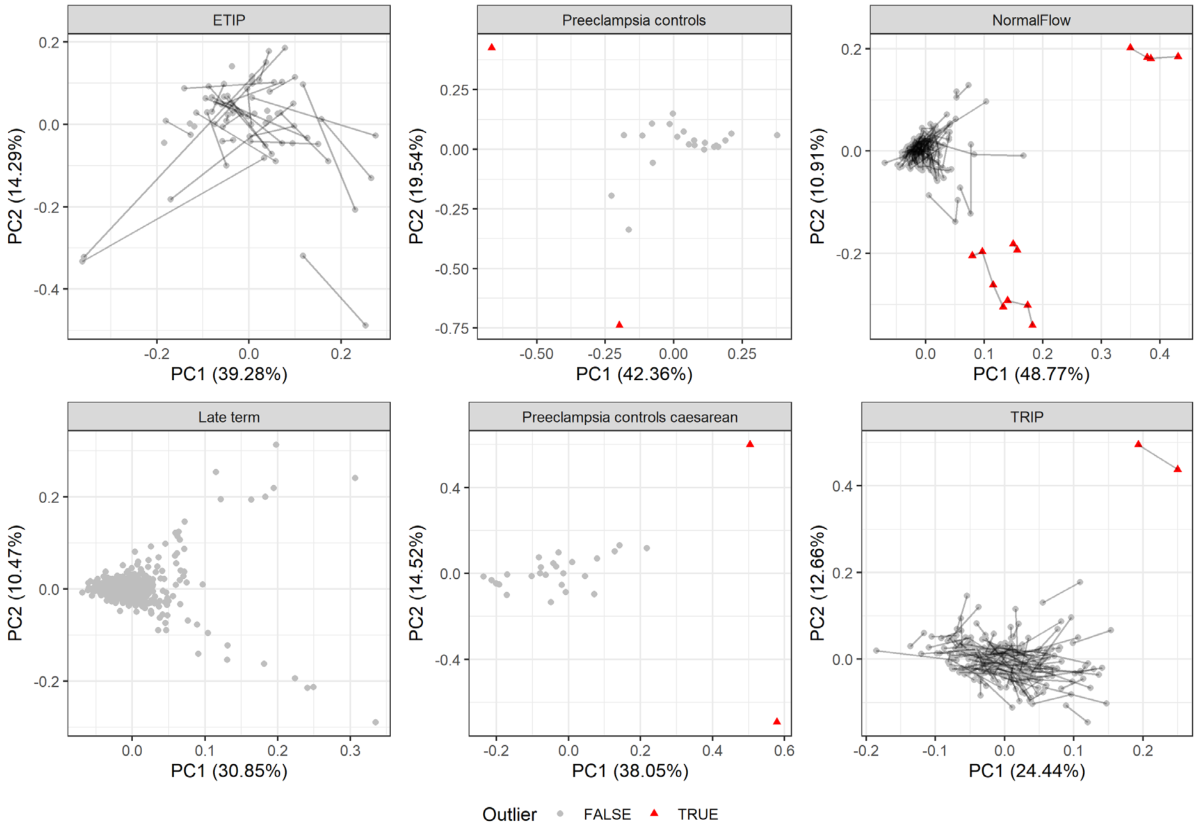


**Supplementary Figure 2**. **Detection of outliers.** Principal component analysis (PCA) was performed by original cohort and inspected for outliers. Outlying samples are indicated as red triangles. Black lines connect samples from the same individual. Abbreviations: PC, principal component. ETIP, Exercise Training in Pregnancy for obese women. TRIP, Training in pregnancy.
