# Supplementary material for: Cytokine Patterns in Maternal Serum From First Trimester to Term and Beyond
Source: Front Immunol. 2021 Oct 14;12:752660. doi: 10.3389/fimmu.2021.752660 (PMC8552528; doi:10.3389/fimmu.2021.752660)
Supplement: Supplementary file 3 [file Image_3.pdf]

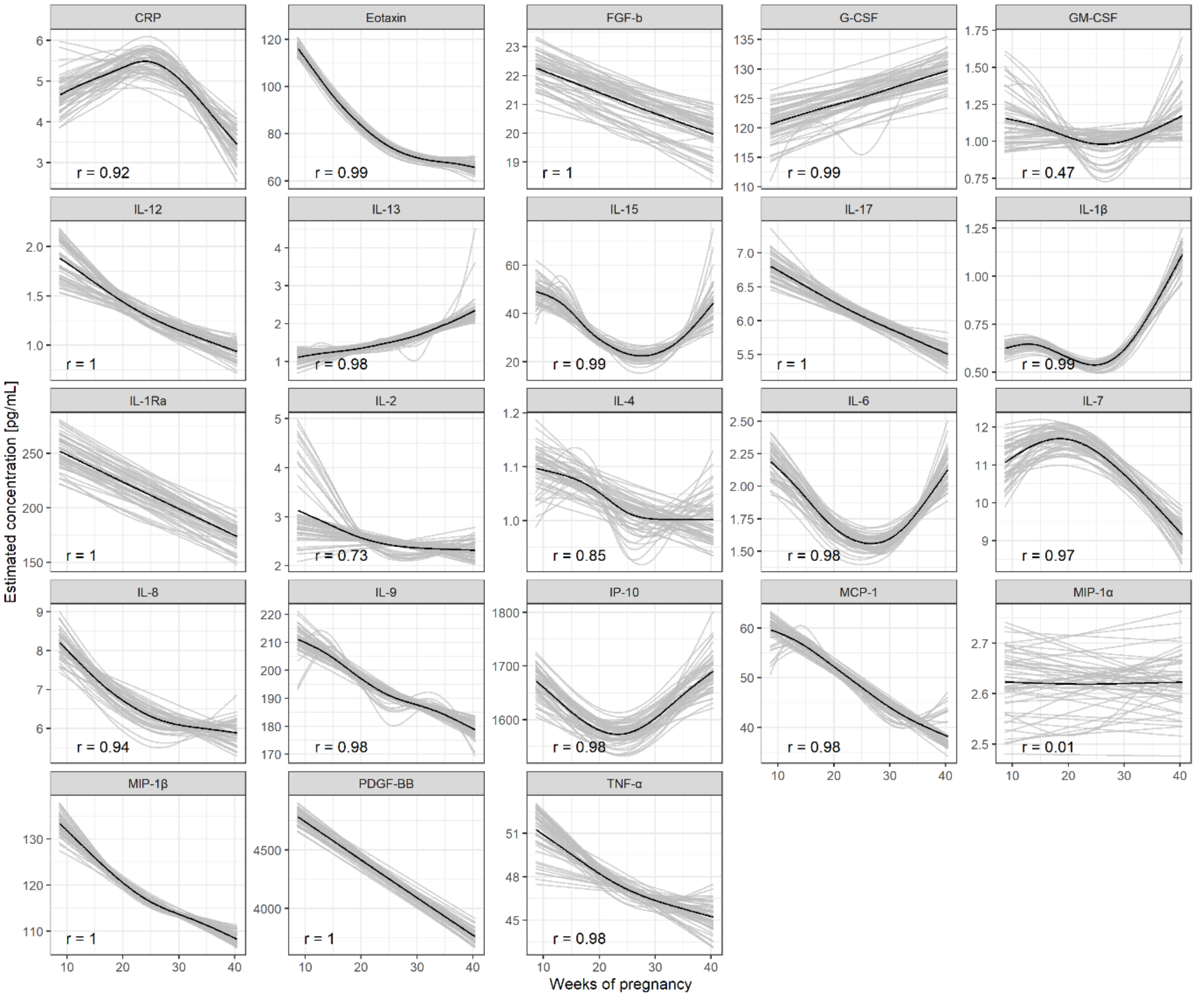

Supplementary Figure 3. **Robustness testing of continuous time trajectories.** Generalized additive mixed models (GAMMs) were built for each cytokine 50 times using 80% of the participants selected at random in each run (grey lines). Spearman's correlation coefficient (*r*) was calculated between each run and the mean value (black line). Cytokines with mean *r* equal to or above 0.9 were considered robust and included for continuous analysis. CRP is measured in µg/mL, cytokines in pg/mL.
