# Supplementary material for: Cytokine Patterns in Maternal Serum From First Trimester to Term and Beyond
Source: Front Immunol. 2021 Oct 14;12:752660. doi: 10.3389/fimmu.2021.752660 (PMC8552528; doi:10.3389/fimmu.2021.752660)
Supplement: Supplementary file 4 [file Image_4.pdf]

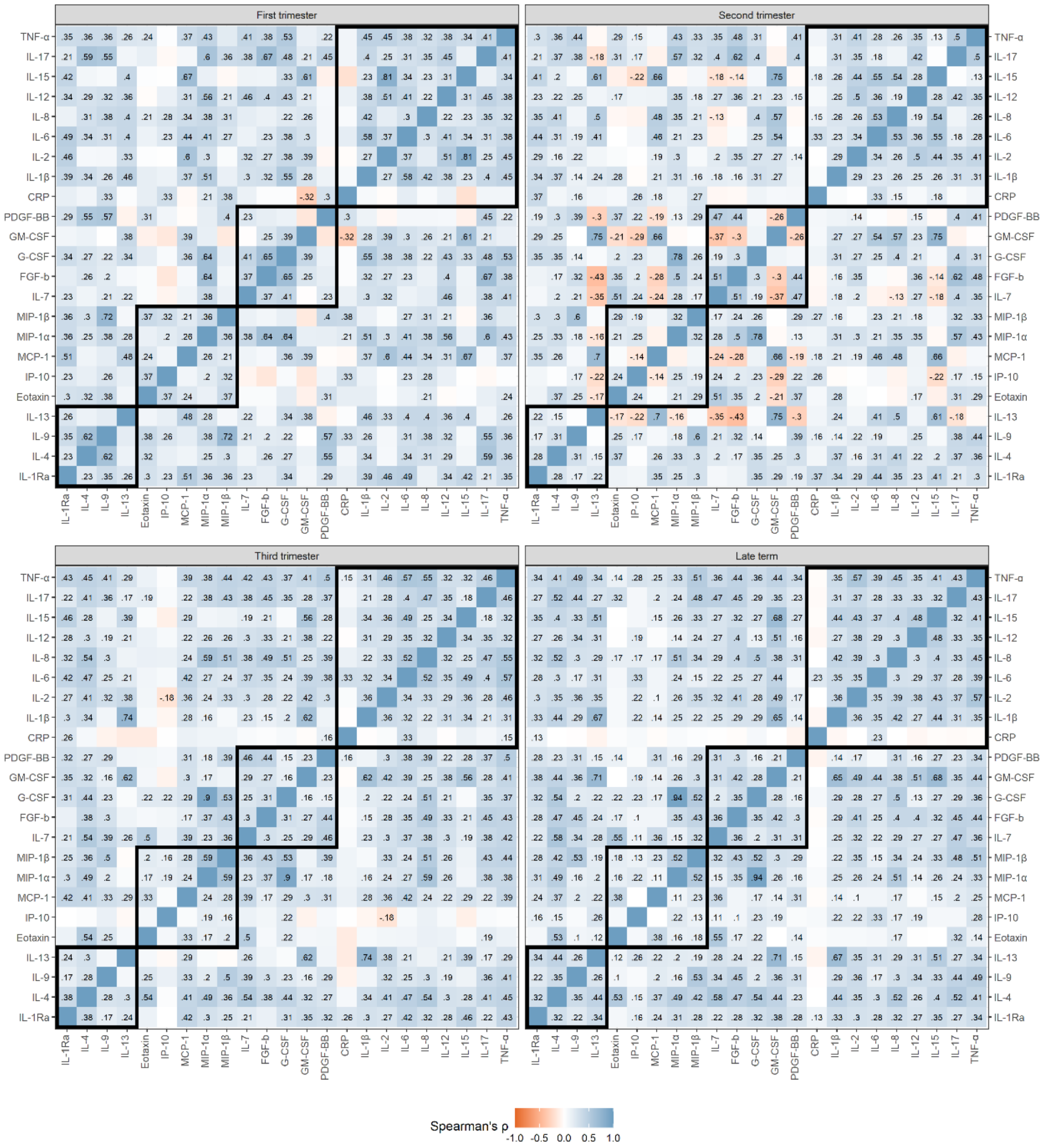

Supplementary Figure 4. Within-trimester correlations for serum cytokine levels. Spearman's correlation coefficient was calculated pairwise between cytokines within each trimester as indicated. Correlation is stated as number when statistically significant ($P < 0.05$). The cytokines are sorted by functional group and the frames enclose within-group correlations.
