# Supplementary material for: Cytokine Patterns in Maternal Serum From First Trimester to Term and Beyond
Source: Front Immunol. 2021 Oct 14;12:752660. doi: 10.3389/fimmu.2021.752660 (PMC8552528; doi:10.3389/fimmu.2021.752660)
Supplement: Supplementary file 5 [file Image_5.pdf]

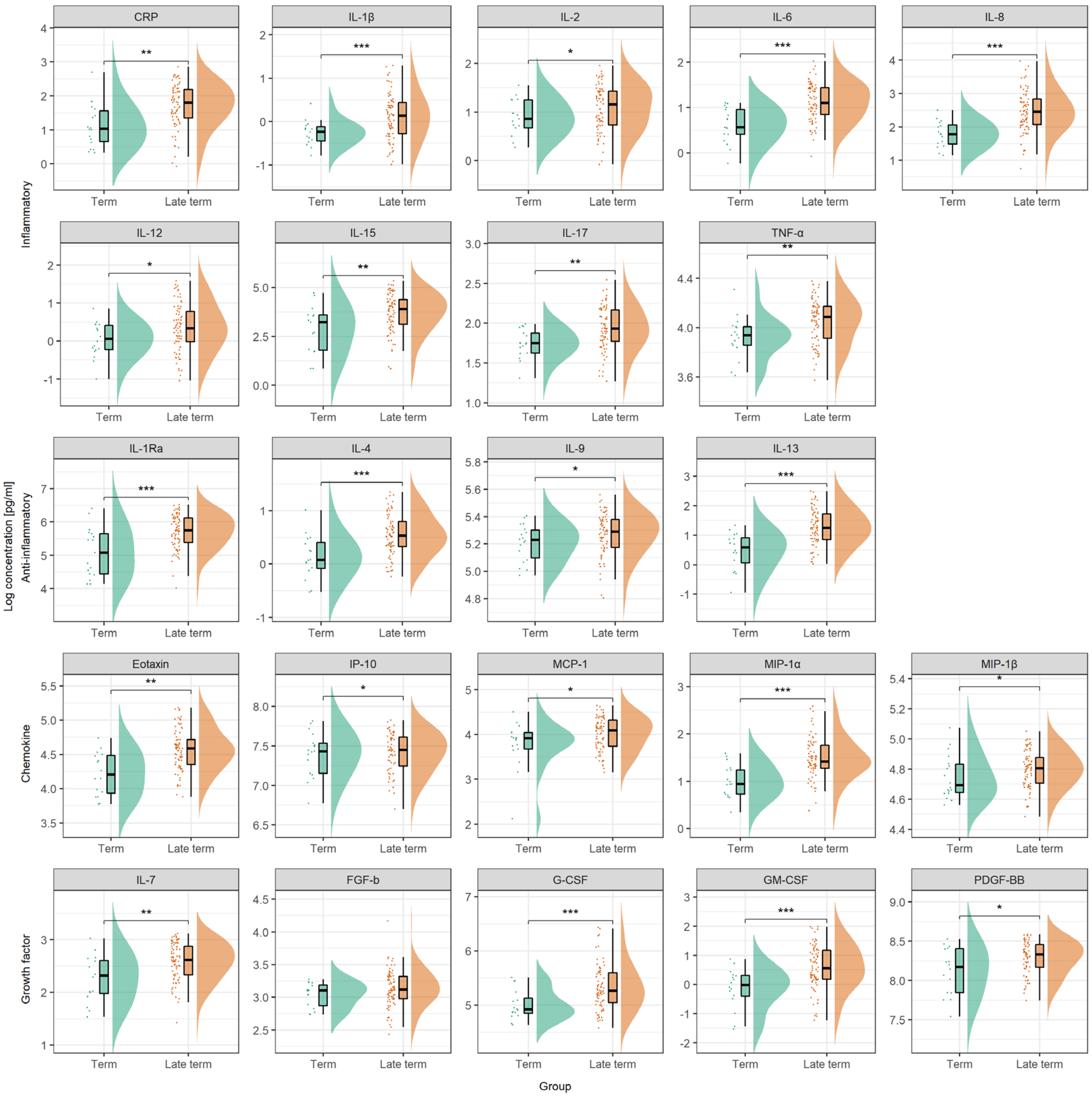

Supplementary Figure 5. Serum cytokine concentration at term and in late term pregnancies with more than four days to delivery. Serum cytokine concentrations are compared between term samples taken between week 37^{+0} and 40^{+3} of pregnancy ($n = 19$) and late term samples taken at approximately week 41^{+2} ($n = 80$). Concentrations are shown as individual measurements, median and quantiles as box plot, and as distribution. To enhance readability, values below the 2.5th and above the 97.5th percentiles are not shown. Statistical significance from Mann-Whitney's U test. CRP is measured in µg/mL, cytokines in pg/mL. $^{*}P < 0.05$, $^{**}P < 0.01$, $^{***}P < 0.001$.
