# Supplementary material for: Cytokine Patterns in Maternal Serum From First Trimester to Term and Beyond
Source: Front Immunol. 2021 Oct 14;12:752660. doi: 10.3389/fimmu.2021.752660 (PMC8552528; doi:10.3389/fimmu.2021.752660)
Supplement: Supplementary file 6 [file Table_1.docx]

**Supplementary Table 1. Detailed cohort information.** General information about the five original cohorts used for selection of pregnant women to the present study.

|  | NormalFlow  (1,2) | ETIP  (3,4) | TRIP  (5,6) | Preeclampsia Study  (7) | Postterm study  (8,9) |
| --- | --- | --- | --- | --- | --- |
| Participants | *n* = 124 | *n* = 91 | *n* = 855 | *n* = 189 | *n* = 508 |
| Study group | Term | Term | Term | Term | Late term |
| Inclusion criteria | (1) Healthy woman, first-trimester pregnancy  (2) Viable, singleton fetus  (3) 18-38 years | (1) Pre-pregnancy BMI ≥ 28 kg/m^2^  (2) Age ≥ 18 years  (3) Live singleton fetus at 11-14 weeks  (4) Gestational age < 18 weeks  (5) Proximity to St. Olavs hospital | (1) Age ≥ 18 years  (2) Live singleton fetus  (3) Proximity to St. Olavs hospital or Stavanger University Hospital | (1) Diagnosed preeclampsia and/or FGR; or  (2) Normal pregnancies with no history of preeclampsia or FGR  (3) Cesarian section  (4) Singleton fetus | (1) Live singleton fetus  (2) Speaking fluent Norwegian  (3) Routine ultrasound scan and delivery at St. Olavs hospital  (4) Cephalic presentation  (5) Gestational age 289 ± 2 days |
| Exclusion criteria | (1) Somatic/mental disease  (2) Complications in earlier pregnancies  (3) Multiple pregnancy  (4) non-Norwegian speaker, long distance to study center  (5) Missed abortions, severe congenital anomalies | (1) High risk for  preterm labor  (2) Diseases that could interfere with participation  (3) Habitual exercise training  (twice or more weekly) in the period before inclusion | (1) High‐risk pregnancies  (2) Diseases that could interfere with participation | (1) Diabetes Mellitus, GDM, gestational hypertension  (2) Trisomy, twin pregnancy | (1) Prelabor rupture of membranes |
| Sampling times | (1) About week 10  (2) About week 12  (3) About week 19  (4) About week 24 | (1) Week 12–18  (2) Week 34–37  (3) Postpartum | (1) Week 18–22  (2) Week 32–36  (3) Postpartum | Prior to delivery week 23-42; and GA-matched samples from normal pregnancies at week 25-42 | Week 41+2 |
| Sampling details | Not fasting  Collected June 2008-May 2010 | Fasting ≥ 10 hour overnight  Collected September 2010-March 2015 | Fasting  Collected April 2007-March 2010 | Not fasting  Collected 2002-2014 | Fasting  Collected September 2002-July 2004 |
| Approvals | REK No. 4.2008.841 | REK No. 2010/1522 and ClinicalTrials.gov (NCT01243554) | REK No. 4.2007.81 and ClinicalTrial.gov (NCT00476567) | REK No. 2012/1040  REK No. 2009/03 | REK No. 106-01 and ClinicalTrial.gov (NCT00385229) |

Abbreviations: FGR, Fetal growth restriction. GDM, Gestational Diabetes Mellitus. GA, gestational age. REK, Regional Committee for Medical and Health Research Ethics. ETIP, Exercise Training in Pregnancy for obese women. TRIP, Training in pregnancy.

# References

1. Stokkeland LMT, Giskeødegård GF, Stridsklev S, Ryan L, Steinkjer B, Tangerås LH, Vanky E, Iversen A-C. Serum cytokine patterns in first half of pregnancy. Cytokine (2019) 119:188–196. doi:10.1016/j.cyto.2019.03.013

2. Stridsklev S, Salvesen Ø, Salvesen KÅ, Carlsen SM, Husøy MA, Vanky E. Uterine artery Doppler measurements during first and second trimesters of normal pregnancy. Acta Obstetricia et Gynecologica Scandinavica (2017) 96:366–371. doi:10.1111/aogs.13073

3. Moholdt TT, Salvesen K, Ingul CB, Vik T, Oken E, Mørkved S. Exercise Training in Pregnancy for obese women (ETIP): study protocol for a randomised controlled trial. Trials (2011) 12:154. doi:10.1186/1745-6215-12-154

4. Garnæs KK, Mørkved S, Salvesen Ø, Moholdt T. Exercise Training and Weight Gain in Obese Pregnant Women: A Randomized Controlled Trial (ETIP Trial). PLOS Medicine (2016) 13:e1002079. doi:10.1371/journal.pmed.1002079

5. Stafne SN, Salvesen K, Romundstad PR, Torjusen IH, Mørkved S. Does regular exercise including pelvic floor muscle training prevent urinary and anal incontinence during pregnancy? A randomised controlled trial. BJOG: An International Journal of Obstetrics & Gynaecology (2012) 119:1270–1280. doi:10.1111/j.1471-0528.2012.03426.x

6. Stafne SN, Salvesen KÅ, Romundstad PR, Eggebø TM, Carlsen SM, Mørkved S. Regular Exercise During Pregnancy to Prevent Gestational Diabetes: A Randomized Controlled Trial. Obstetrics & Gynecology (2012) 119:29–36. doi:10.1097/AOG.0b013e3182393f86

7. Stødle GS, Silva GB, Tangerås LH, Gierman LM, Nervik I, Dahlberg UE, Sun C, Aune MH, Thomsen LCV, Bjørge L, et al. Placental inflammation in pre‐eclampsia by Nod‐like receptor protein (NLRP)3 inflammasome activation in trophoblasts. Clinical & Experimental Immunology (2018) 193:84–94. doi:10.1111/cei.13130

8. Heimstad R, Skogvoll E, Mattsson L-Å, Johansen OJ, Eik-Nes SH, Salvesen KÅ. Induction of Labor or Serial Antenatal Fetal Monitoring in Postterm Pregnancy: A Randomized Controlled Trial. Obstetrics & Gynecology (2007) 109:609–617. doi:10.1097/01.AOG.0000255665.77009.94

9. Carlsen SM, Heimstad R. Androgen levels are associated with blood pressure in pregnant women after term. Acta Obstetricia et Gynecologica Scandinavica (2012) 91:232–236. doi:10.1111/j.1600-0412.2011.01280.x
