# Supplementary material for: Cytokine Patterns in Maternal Serum From First Trimester to Term and Beyond
Source: Front Immunol. 2021 Oct 14;12:752660. doi: 10.3389/fimmu.2021.752660 (PMC8552528; doi:10.3389/fimmu.2021.752660)
Supplement: Supplementary file 7 [file Table_2.docx]

**Supplementary Table 2. Immunoassay performance.** Median serum concentration in pg/mL, lower (L-) and upper (U-) limit of quantification (LOQ) in pg/mL, and number of samples above ULOQ/below LLOQ. All serum samples analyzed in this batch are included below, prior to any sample exclusion or replacement of missing values (1166 serum samples). Serum samples from the NormalFlow study were analyzed in relation to a previous study and are reported elsewhere (1).

|  | Median (25.-75. percentile) | LLOQ, ULOQ | <LLOQ, *n* (%) | > ULOQ, *n* (%) |
| --- | --- | --- | --- | --- |
| IL-1β | 0.9 (0.6-1.4) | 0.1, 405.3 | 28 (2) | 0 (0) |
| IL-1Ra | 302.7 (187.7-454.9) | 35.8, 13595.2 | 81 (7) | 0 (0) |
| IL-2 | 3.0 (2.1-4.2) | 0.5, 2836.7 | 13 (1) | 0 (0) |
| IL-4 | 1.4 (1.0-2.0) | 0.2, 316.3 | 6 (1) | 0 (0) |
| IL-5 | 4.6 (2.8-8.0) | 1.4, 7312.5 | 230 (20) | 0 (0) |
| IL-6 | 2.5 (1.6-3.9) | 0.1, 586.1 | 2 (0) | 0 (0) |
| IL-7 | 13.4 (10.3-17.2) | 1.3, 3160.9 | 1 (0) | 0 (0) |
| IL-8 | 9.1 (6.0-14.2) | 0.7, 1418.9 | 10 (1) | 0 (0) |
| IL-9 | 198.7 (179.6-216.9) | 14.7, 1513.4 | 1 (0) | 4 (0) |
| IL-10 | 1.4 (0.8-2.4) | 0.4, 2044.0 | 838 (72) | 0 (0) |
| IL-12 | 1.4 (0.9-2.2) | 0.4, 2440.7 | 104 (9) | 0 (0) |
| IL-13 | 2.4 (1.5-4.4) | 0.4, 495.3 | 55 (5) | 0 (0) |
| IL-15 | 52.3 (32.2-91.7) | 14.8, 8886.2 | 279 (24) | 0 (0) |
| IL-17 | 6.7 (5.4-8.2) | 0.8, 4109.7 | 0 (0) | 0 (0) |
| Eotaxin | 88.7 (68.3-113.7) | 0.3, 243.6 | 0 (0) | 9 (1) |
| FGF-b | 22.2 (17.6-26.5) | 2.0, 2050.6 | 3 (0) | 0 (0) |
| G-CSF | 157.4 (110.2-220.4) | 2.7, 7634.5 | 0 (0) | 1 (0) |
| GM-CSF | 1.5 (0.9-2.6) | 0.3, 486.6 | 57 (5) | 0 (0) |
| IFN- γ | 9.3 (3.1-22.3) | 0.9, 1968.0 | 1071 (92) | 0 (0) |
| IP-10 | 1623.5 (1259.8-2028.8) | 11.2, 2740.0 | 2 (0) | 156 (13) |
| MCP-1 | 53.8 (38.8-73.5) | 0.3, 622.2 | 0 (0) | 1 (0) |
| MIP-1α | 3.5 (2.5-4.8) | 0.3, 53.9 | 48 (4) | 5 (0) |
| MIP-1β | 118.8 (108.5-129.7) | 3.0, 577.9 | 2 (0) | 0 (0) |
| PDGF-BB | 4284.8 (3528.0-4956.9) | 11.3, 5851.8 | 0 (0) | 253 (22) |
| RANTES | - | 6.7, 1403.5 | 2 (0) | 1164 (100) |
| TNF-α | 46.1 (54.6-63.4) | 1.4, 4694.3 | 1 (0) | 0 (0) |
| VEGF | 117.8 (58.8-244.4) | 19.8, 8665.3 | 1030 (88) | 0 (0) |

# References

1. Stokkeland LMT, Giskeødegård GF, Stridsklev S, Ryan L, Steinkjer B, Tangerås LH, Vanky E, Iversen A-C. Serum cytokine patterns in first half of pregnancy. Cytokine (2019) 119:188–196. doi:10.1016/j.cyto.2019.03.013
